# Integration of intermittent calcium signals in T cells revealed by temporally patterned optogenetics

**DOI:** 10.1016/j.isci.2023.106068

**Published:** 2023-01-26

**Authors:** Béatrice Corre, Yassine El Janati Elidrissi, Justine Duval, Mailys Quilhot, Gaëtan Lefebvre, Solène Ecomard, Fabrice Lemaître, Zacarias Garcia, Armelle Bohineust, Erica Russo, Philippe Bousso

**Affiliations:** 1 Dynamics of Immune Responses Unit, Institut Pasteur, Université Paris Cité, Inserm U1223, 75015 Paris, France

**Keywords:** Biological sciences, Biochemistry, Immunology, Immunological methods

## Abstract

T cells become activated following one or multiple contacts with antigen-presenting cells. Calcium influx is a key signaling event elicited during these cellular interactions; however, it is unclear whether T cells recall and integrate calcium signals elicited during temporally separated contacts. To study the integration of calcium signals, we designed a programmable, multiplex illumination strategy for temporally patterned optogenetics (TEMPO). We found that a single round of calcium elevation was insufficient to promote nuclear factor of activated T cells (NFAT) activity and cytokine production in a T cell line. However, robust responses were detected after a second identical stimulation even when signals were separated by several hours. Our results suggest the existence of a biochemical memory of calcium signals in T cells that favors signal integration during temporally separated contacts and promote cytokine production. As illustrated here, TEMPO is a versatile approach for dissecting temporal integration in defined signaling pathways.

## Highlights

• Temporally patterned optogenetics (TEMPO) interrogates signal integration in cells
• TEMPO relies on a programmable device that runs multiple photoactivation patterns
• TEMPO revealed that T cells integrate calcium signals received several hours apart
• Integration of calcium signals promotes NFAT activity and cytokine production

## Graphical abstract

**Figure:**
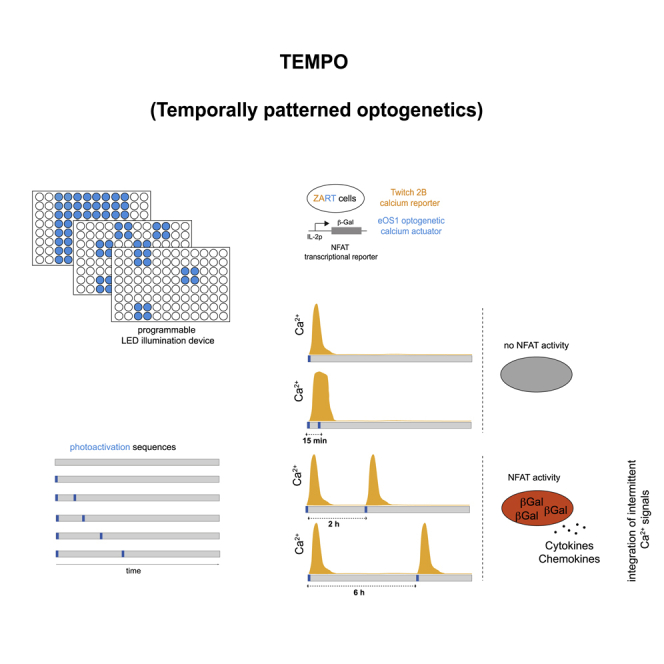

## Introduction

T lymphocytes are highly motile cells that become activated following one or successive encounters with antigen-presenting cells (APCs).^1^^,^^2^ A key signaling event during antigen recognition by T cells is the elevation of intracellular calcium concentration^3^ and subsequent activity of the nuclear factor of activated T cells (NFAT) family of transcription factors which are essential for proper T cell activation and play an important role for the induction of effector functions such as cytokine/chemokine production.^4^^,^^5^ Previous studies that used Ca^2+^ clamp technique or pharmacological manipulations have shown that Ca^2+^ oscillations and duration can trigger distinct patterns of gene expression.^6^^,^^7^ It is however unclear whether T cells have the ability to recollect calcium signals over longer periods, for example, after sequential interactions with APCs several hours apart.

Optogenetics represents a powerful approach to interrogate the consequence of specific signaling pathways or to decode how cells interpret signals of different duration and frequency. For example, a light-controlled Ras has been used to assess signal transmission to the MAPK Erk and to reveal that distinct patterns of Ras signaling elicited distinct functional outcomes.^8^ A similar strategy was used to dissect how oncogenic mutations could alter the interpretation of Ras signals.^9^ In different studies, optogenetic manipulation uncovered how Erk dynamics alter cell proliferation^10^ or interrogated the impact of input pulses on downstream signal integration.^11^ Optogenetic control of transcription factor translocation also revealed distinct gene-specific interpretation of nuclear translocation dynamics.^12^ Applied to calcium signals in HeLa cells, optogenetic manipulations supported the idea that NFAT activation is more sensitive to cumulative signals than to oscillation frequency.^13^

Here, we developed TEMPO (temporally patterned optogenetics), an approach consisting of a dedicated device (TEMPO device) for high-throughput assessment of photoactivation patterns in 96-well microplates and an easy-to-use graphical user interface to program up to 18 photoactivation sequences. We used the calcium actuator enhanced OptoSTIM1 (eOS1)^14^ to interrogate the rules of calcium signal integration in a T cell line. We report the capacity of T cells to integrate signals received several hours apart to promote NFAT activity and to elicit production of multiple cytokines and chemokines.

## Results

### TEMPO: A dedicated device for programmable, multiplexed photoactivation sequences

**Figure 1:**
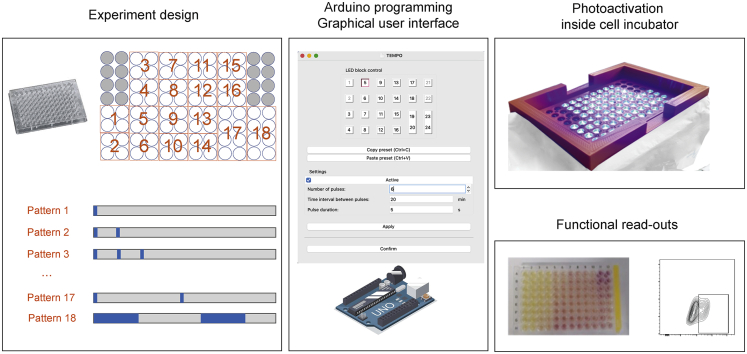
TEMPO: a dedicated approach for programmable, multiplexed photoactivation sequences TEMPO (temporally patterned optogenetics) is an approach designed to characterize the impact of a multiple photoactivation sequences on cells expressing specific optogenetic actuators. The experimental design includes the plating of cells expressing an optogenetic actuator of interest in 96-well plates and the programming of up to 18 photoactivation sequences (controlling pulse durations and intervals) with a simple-to-use graphical interface. Blue-LED illumination is performed directly in the cell incubator. After the photoactivation and additional culture period if needed, cells and supernatants can be analyzed using a variety of assays including colorimetric assays, flow cytometry, or ELISA.

Optogenetics represents a technique of choice to manipulate signaling pathways. To facilitate and multiplex photoactivation sequences, we thought to design a blue LED-based illumination device for temporally patterned optogenetics (TEMPO device) and a dedicated graphical user interface for programming photoactivation sequences (Figure 1). The support was 3D printed and designed to support conventional 96-well culture plates. Controlled switches, piloted by an Arduino card, were used to control groups of 4 or 8 neighboring LEDs operated by an external power supply. A total of 18 groups of LEDs (16 groups with 4 replicate wells and 2 groups with 8 replicate wells) could be independently programmed using the graphical user interface to provide pulses of light of desired number and duration and repeated at desired intervals (Figure 1). The 18 outputs were obtained by using the original 13 outputs of the Arduino card and converting 5 inputs into outputs. After programming, the device can hold a standard 96-well microplate containing cells of interest and can be placed in conventional cell incubators for hours or days to deliver light with temporally defined patterns in each independent group of LEDs (Figure 1).

### The duration of calcium signals controls NFAT activity in a T cell hybridoma

**Figure 2:**
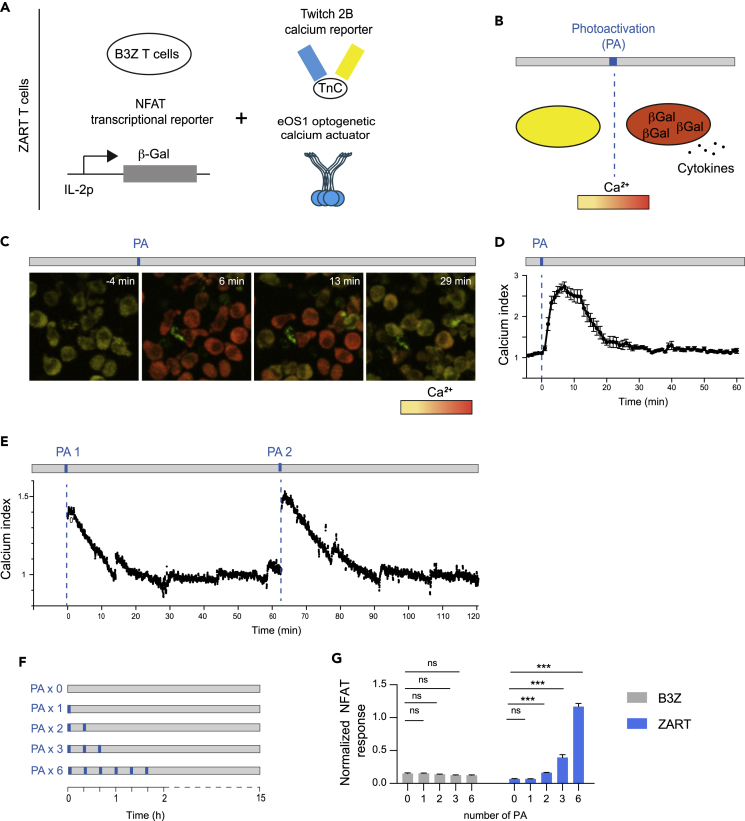
Defining the cellular response to photoactivation using the eOS1 calcium actuator (A) Generation of ZART T cells. The B3Z T cell hybridoma (expressing β−Galactosidase under the control of the NFAT elements of the IL-2 promoter) was retrovirally transduced to express the FRET-based calcium reporter Twitch2B and the eOS1 (enhanced OptoSTIM-1) calcium actuator. (B) Schematic representation of the consequence of photoactivation in ZART T cells, including calcium elevation, β-galactosidase activity, and cytokine production. (C and D) The calcium response triggered by a single round of photoactivation in ZART T cells was evaluated using live imaging using two-photon excitation. (C) Representative time-lapse images showing the kinetics of intracellular calcium elevation in ZART T cells following a 5s LED illumination using the TEMPO device. (D) The mean calcium signal was quantified over time, 5 min before and up to 60 min after photoactivation. Data are representative of 2 independent experiments. (E) Monitoring the calcium response after repeated photoactivation. ZART T cells were subjected to a single photoactivation (5s pulse) or two rounds of photoactivation (5s pulses) 1 h apart using TEMPO. Cell aliquots were collected at various time points after each photoactivation and analyzed by time-resolved flow cytometry to monitor calcium levels using the Twitch2B fluorescent reporter. Each cell aliquot was acquired for 15 min and immediately replaced by a new aliquot to generate an almost continuous signal curve by concatenation. Aliquot switching can results in minor changes in calcium index. Note that the responses after the first or the second photoactivation are similar in intensity and duration. Data are representative of 3 independent experiments. (F and G) Evaluating the minimal duration of calcium signal to elicit NFAT transcriptional activity in ZART T cells. (F) ZART T cells or control B3Z T cells were subjected to TEMPO using from 0 to 6 rounds of photoactivation every 20 min. (G) After 15 h, β-galactosidase activity was quantified in the cell lysate. Some ZART T cells were treatd with thapsigargin during the whole assay to estimate the maximal response and used to normalize the data (expressed as % of maximal response). Data are representative of 4 independent experiments.

As a cell model system to manipulate calcium signals, we used the B3Z T cell hybridoma that expressed NFAT-LacZ reporter gene.^15^ B3Z T cells were also retrovirally transduced to express the optogenetic calcium actuator eOS1,^14^ an enhanced version of the Cry2-based OPTOStim1 actuator.^16^ In this system, Cry2-Olig-mediated oligomerization of the calcium sensor STIM1 upon blue light exposure results in opening of the CRAC channels and subsequent calcium influx in the cell. In addition, B3Z T cells were transduced to express Twitch2B, a fluorescence resonance energy transfer-based calcium reporter.^17^ Our modified B3Z T cells therefore offer the possibility to manipulate calcium signals with light, to monitor calcium levels in real time and to assay NFAT transcriptional activity or cytokine production (Figures 2A and 2B). These cells will be referred thereafter as ZART T cells for B3Z T cells with calcium Actuator, Reporter, and Transcriptional reporter (Figures 2A and 2B).

We first set-out to define the calcium response in ZART cells after a single pulse of blue light (5s) using our LED-based device. Using real-time imaging, we observed an almost instantaneous rise in intracellular calcium in virtually all photoactivated cells (Figure 2C). As shown in Figures 2C and 2D, calcium levels returned to baseline after 20–30 min. Such transient calcium elevation is within physiological range of what could be observed during certain T cell APC contacts *in vivo.*^18^^,^^19^ We confirmed this kinetic by tracking calcium signals using time-resolved flow cytometry (Figure 2E). Of note, when cells were subjected to a second photoactivation 1 h after the initial stimulation, we observed a similar pattern of calcium response (typically lasting 20–30 min). We also subjected the cells to two photoactivations separated by only 15 min and observed the expected prolonged duration (total of 40–45 min) of calcium elevation (Figure S1). Thus, it is possible to generate repeated and defined patterns of calcium elevation using specific pulses of blue light (Figure 2E).

Having defined the duration of calcium elevation provoked by a single light pulse, we tested the minimum duration of calcium signaling for effective NFAT activity. We used TEMPO to provide repeated pulses (from 1 to 6 pulses) of calcium every 20 min to generate an almost continuous calcium signal of defined duration (Figure 2F). We monitored the NFAT transcriptional response by measuring β-galactosidase activity in lysed cells at 15 h. Interestingly, a single photoactivation (thus representing a 20 min period of elevated calcium) was insufficient to elicit NFAT activity (Figure 2G). We noted that a minimum of three photoactivations corresponding to an estimated 1-h period of calcium elevation was required for robust NFAT transcriptional activity (Figure 2G).

### ZART T cells integrate calcium signals triggered by temporally separated stimulations

**Figure 3:**
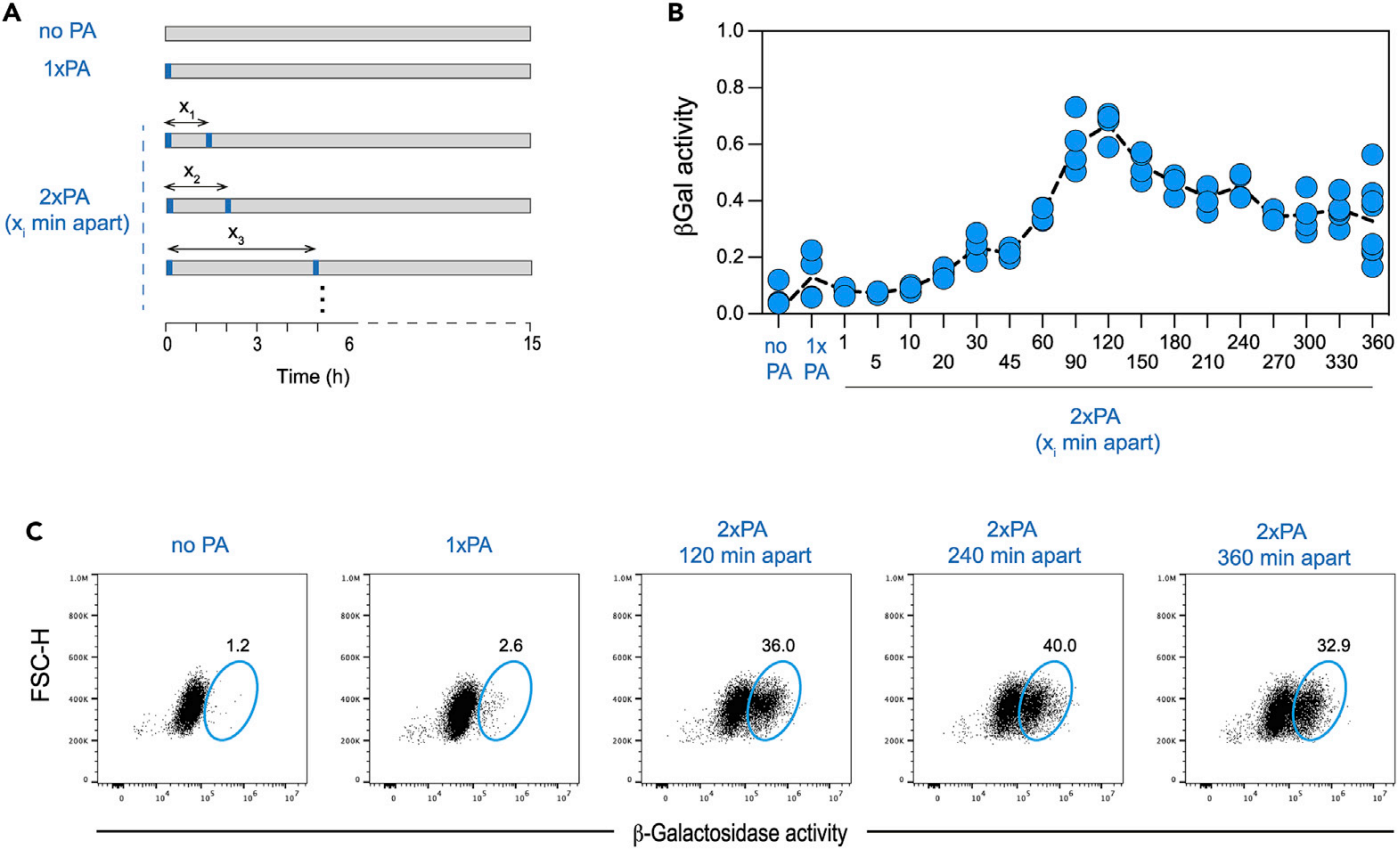
Cells can integrate calcium signals separated by several hours ZART T cells were subjected to TEMPO to evaluate their capacity to integrate intermittent calcium signals. Cells were exposed (or not) to one or two pulses (5s) of photoactivation delivered with the indicated time interval. After 15 h, β-galactosidase activity was quantified in the cell supernatant. (A) Experimental set-up illustrating the different sequences of photoactivation programmed in the TEMPO device. (B) Bulk NFAT transcriptional responses were evaluated by monitoring β-galactosidase activity in cell lysates for the indicated photoactivation sequences. (C) Single-cell assessment of NFAT transcriptional activity using the flow cytometric FDG assay to reveal β-galactosidase activity. Results are representative of 4 independent experiments.

Having defined the approximate duration of calcium signals required for NFAT activity and established that one photoactivation is insufficient to elicit NFAT activity, we asked whether two suboptimal, temporally separated periods of calcium elevation could generate an NFAT response. We used the TEMPO to deliver two pulses of photoactivation separated by distinct periods of time (ranging from 1 min to 6 h) and assayed NFAT activity on cell lysates at 15 h (Figure 3A). Again, a single stimulation was not sufficient to elicit NFAT activity. No activity could also be detected if the two stimulations were too close together (less than 30 min), possibly reflecting the minimal duration of calcium signal needed for NFAT activity (Figure 3B). However, two photoactivations separated by 1 h or more were enough to promote robust NFAT responses (Figure 3B). Strong NFAT responses were observed even when the two stimulations were given 6 h apart. As shown in Figure S2, very similar results were obtained when the readout was performed 3 h after the last stimulation for individual conditions (instead of a fixed time point of 15 h), highlighting the robustness of the observations. We also repeated these observations using a second ZART T cell clone (Figure S2). In addition, we confirmed during the course of these experiments the absence of detectable optical signal leakage in adjacent wells by analyzing non-illuminated wells adjacent to stimulated wells (Figure S3). We also analyzed NFAT activity at the single-cell level using flow cytometry and similarly observed that suboptimal calcium responses given apart could be productively integrated (Figure 3C). Analyzing longer intervals between the two photoactivations, we found that signal integration remained detectable in all conditions although the efficiency slowly decayed for time intervals longer than 6 h (Figure S4). These results provide evidence that T cells can maintain a biochemical memory of past calcium responses favoring the integration of suboptimal stimulations at the scale of hours.

### Integration of intermittent calcium signals promotes cytokine production

Cytokine production is an important effector function of T cells and is largely triggered by antigen recognition. To test if calcium signals alone can be sufficient to trigger the release of certain cytokines or chemokines, we stimulated murine primary effector T cells with either the calcium ionophore ionomycin or with thapsigargin for 6 h. As shown in Figure S5, both ionomycin and thapsigargin treatment led to the production of several cytokines/chemokines including CCL3, CCL4, CCL5, IL-18, and IFN-γ.

**Figure 4:**
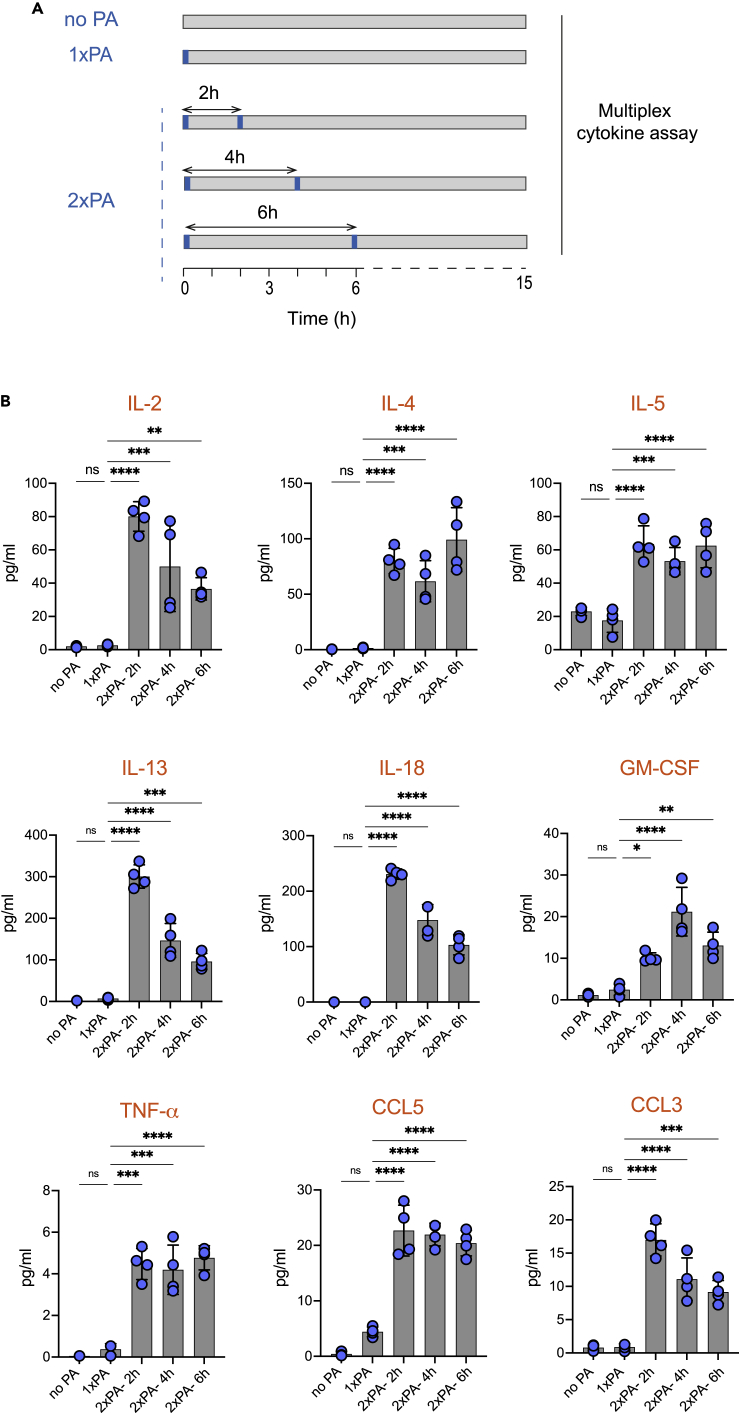
Integration of intermittent calcium signals promotes the production of multiple cytokines ZART T cells were subjected to TEMPO to evaluate cytokine production following intermittent calcium signals. Cells were exposed (or not) to one or two pulses (5s) of photoactivation delivered with the indicated time interval. After 15 h, cell supernatants were collected and subjected to a multiplex cytokine assay. (A) Experimental set-up illustrating the different sequences of photoactivation programmed in the TEMPO device. (B) Cytokine/chemokine (IL-2, IL-4, IL-5, IL-13, IL-18, GM-CSF, TNF-α, CCL5, and CCL3) concentrations measured with the indicated patterns of photoactivation. Results are representative of 2 independent experiments. ∗∗, p < 0.01; ∗∗∗, p < 0.001; ns, not significant (one-way ANOVA).

To test if the rule underlying calcium signals integration can result in cytokine production, we used TEMPO to deliver two pulses of photoactivation separated by distinct periods of time (ranging from 2 to 6 h) to ZART T cells and collected cell supernatants at 15 h (Figure 4A). A multiplex cytokine assay was conducted to detect the presence of 20 cytokines or chemokines simultaneously. We found that a single photoactivation did not lead to cytokine/chemokine release; however, we detected multiple cytokines (9/20 tested) after two stimulations spaced 2 to 6 h apart. This was the case for IL-2, IL-4, IL-5, IL-13, IL-18, GM-CSF, TNF-α, CCL3, and CCL5 (Figure 4B). The other cytokines tested (IL-6, IL-1β, IL-12p70, IFN-g, CCL11, CXCL1, CCL10, CCL2, CCL4, CCL7, and CXCL2) were not detected upon ZAR T cells photoactivation. These results strongly suggest that T cells have the capacity to integrate temporally separated calcium signals to release multiple cytokines and chemokines.

## Discussion

Optogenetics is a powerful approach to decode how cells integrate stimulations and signaling events that can differ in duration and frequency. Here, we developed TEMPO, an optogenetic-based approach that uses a custom-made LED-based illumination device and a dedicated graphical user interface for multiplexing photoactivation sequences. Together with recently developed optogenetic devices,^9^^,^^13^^,^^20^^,^^21^ our system is compatible with standard multi-well culture plates. Key features of TEMPO include the capacity to run up to 18 independent photoactivation sequences in parallel with multiple replicates and the use of a very user-friendly graphical interface that does not require programming skills for selecting illumination patterns.

One striking observation made using TEMPO is the T cell ability to integrate two calcium signals (inefficient individually) delivered hours apart to induce robust NFAT transcriptional activity and the production of a wide array of cytokines and chemokines. This property may contribute to the previous observation that T cells can integrate suboptimal T cell receptor (TCR) stimulations to proliferate.^22^^,^^23^ Our results also suggest that integration of repeated stimulations (for example, in the context of short-lived T cell APC interactions) may play a major role for release of cytokines and chemokines. Of note, the lifetime of dephosphorylated NFAT can contribute to integrate Ca^2+^ oscillations but only when intervals are shorter than 5–10 min.^24^ The slow nuclear export of NFAT (t_1/2_ of 20 min) was also visualized *in vivo* and constituted a short-term memory of TCR signals.^25^ A signal persistence of intracellular signals after TCR stimulation was also shown to be in the range of 15 min.^26^ In addition to these relatively short-term memory effects, we described here a long-term memory of calcium signaling (up to 6 h), hence most likely accounted for by a different mechanism. Future applications of TEMPO will help clarify the molecular basis of signal integration in T cells but could also provide a mean to optimize activation sequences to generate the most effective T cells/chimeric antigen receptor T cells for adoptive cell therapy.

### Limitations of the study

Limitations of our approach include the use of fixed light intensity and the use of a single wavelength for photoactivation. The TEMPO device is also primarily compatible with 96-well microplates but not with other formats.

## STAR★Methods

### Key resources table

**Table:** 

REAGENT or RESOURCE	SOURCE	IDENTIFIER
**Bacterial and virus strains**		

pMSCV-Twitch2B	This paper	N/A
pMSCV-eOS1mScarlet	Bohineust et al. 2020^14^	N/A

**Chemicals, peptides, and recombinant proteins**		

chlorophenol red-β-D-galactopyranoside	Roche	10884308001

**Critical commercial assays**		

Venor®GeM Advance	Minerva Biolabs GmbH	11–7024
FluoReporter™ lacZ Flow Cytometry Kit	ThermoFisher Scientific	F1930
Th1/Th2 Cytokine & Chemokine 20-Plex Mouse ProcartaPlex™ Panel 1	ThermoFisher Scientific	EPX200-26090-901

**Deposited data**		

TEMPO software	https://github.com/boussolab/TEMPO	N/A
3D printing files	https://github.com/boussolab/TEMPO	N/A

**Experimental models: Cell lines**		

B3Z T eOS1-mScarlet	Bohineust et al. 2020^14^	N/A
HEK-293T	ATCC	CRL-3216

**Recombinant DNA**		

pcDNA3-Twitch2B	Bohineust et al., 2020^14^	N/A

**Software and algorithms**		

FlowJo v10.8.0	BD Biosciences	N/A
Fiji (ImageJ 2.1.0)	Schindelin et al. 2012^27^	https://fiji.sc
Imaris 7.4.2 software	Oxford Instruments	N/A
Bio-Plex Manager 6.2	Bio-Rad	N/A
TEMPO software	This paper	N/A

### Resource availability

#### Lead contact

Further information and requests for resources and reagents should be directed to and will be fulfilled by the lead contact, Philippe Bousso (philippe.bousso@pasteur.fr).

#### Materials availability

Newly generated materials are available upon request to the lead contact.

### Experimental models and subject details

#### Cell lines

To generate ZART cells, B3Z T cell hybridomas^15^ containing the LacZ reporter gene expressed under the control of NFAT elements of the IL-2 promoter were retrovirally transduced to express the calcium reporter Twitch2B^17^ and the enhanced OptoSTIM1 (eOS1) actuator fused to mScarlet.^14^ After transduction, cells were cloned and individual clones were tested for their response to photoactivation. Two clones were selected for the study. ZART T cells were routinely tested for the absence of *Mycoplasma* contamination (Venor-GeM Advance mycoplasma detection kit, Minerva Biolabs). Cells were cultured in complete RPMI.

### Method details

#### Design of the TEMPO device and software

A 96-well culture plate holder was designed using the online software OnShape and 3D-printed. Ultrabright blue LEDs (470 nm, #C503B-BCS-CV0Z0461, Farnell) were arranged on an electronic plate and connected by blocks of 4. Each block of 4 LEDs was soldered with an analog switch (16 pins, #DG201BDJ PDIP, RS Component), connected to one of the outputs of an Arduino board. Four blocks of LEDs were not connected for negative (non-photoactivated) controls in the 96-well plate. A tropicalization coating was applied to protect the components from the humidity. A protective box was built using laser cutting to protect all electronic components. To program photoactivation sequences, a dedicated software with a friendly graphical user interface was developed in Python using the tkinter library. The software allows to assign to each individual LED block a photoactivation sequence defined by the pulse number, the pulse duration and the interval between pulses. The TEMPO software can establish a direct connection with the TEMPO device in order to upload photoactivation sequences onto the Arduino board.

#### *In vitro* live imaging of calcium signals

ZART T cells were cultured in complete RPMI without phenol red supplemented with 2% heat-inactivated fetal bovine serum, 50 U mL^−1^ penicillin, 50 μg mL^−1^ streptomycin, 1 mM sodium pyruvate, 10 mM HEPES and 50 μM 2-mercaptoethanol and maintained at 37°C. Cells were placed in a petri dish above the TEMPO device. *In vitro* two-photon imaging of calcium signals was performed with an upright microscope (FVMPE-RS, Olympus), a 25×/1.05 numerical aperture, water-dipping objective and using FV31S-SW software (Olympus). Excitation was provided by an Insight DeepSee dual laser (Spectra-Physics) tuned at 880 nm and image acquisition was performed using a resonant scanner (0.067 μs/pixel, 10 integrations). The following filters were used for fluorescence detection CFP:483/32; FRET: 542/27. Photoactivation was performed with the TEMPO device using a 5s pulse of blue-LED illumination. Time-lapse movies were processed and analyzed using Fiji.^27^ Calcium was calculated after cell tracking (surface tracking mode, Imaris 7.4.2 software) by dividing FRET signals by CFP signals in individual cells.

#### Calcium measurements by flow cytometry

ZART T cells were cultured in flat-bottom 96-well plates (at 3x10^5^ cells/well) and subjected to a single photoactivation (5s pulse) or two rounds of photoactivation (5s pulses) 1 h apart using TEMPO. Cells were maintained in the incubator at 37°C and individual wells were collected at various time points after each photoactivation and analyzed by time-resolved flow cytometry using a Cytoflex LX (Beckman Coulter) flow cytometer to monitor calcium levels based on the Twitch2B reporter. Each cell aliquot was acquired for 15 min and immediately replaced by a new aliquot to generate an almost continuous signal curve by concatenation. The calcium index was obtained by dividing the FRET signal (filter 550/30) by the CFP signal.

#### Bulk and single-cell measurement of NFAT activity

To test NFAT transcriptional activity, ZART T cells cultured in 96-well plates (3x10^5^ cells/well) were subjected to various sequences of photoactivation using TEMPO and subsequently kept in culture for a total period of 15h. For bulk measurements, cells were washed twice in phosphate-buffered saline (PBS) and lysed in 100 μL per well of CPRG buffer (PBS + 9 mM MgCl_2_ + 0.125% NP40 + 100 μm β-mercaptoethanol + 0.15 mM chlorophenol red-β-D-galactopyranoside (Roche, #10884308001). Plates were incubated in the dark at room temperature for 30 min to 1 h and the optical density was read at 570 nm (reading at 620 nm was used as reference and subtracted). Cells treated with thapsigargin (1 μM) were used as a positive control and when indicated for normalizing the data. For flow cytometric analyses of NFAT activity, ZART T cells were resuspended in flow cytometer tubes in 50 μL at the end of the experiment, loaded with fluorescein di β-D-galactopyranoside (FDG) at 37°C by hypotonic shock using manufacturer instructions (FluoReporter ® lacZ Flow Cytometry Kit, Invitrogen). FDG loading was stopped after 1 min by adding 0.9 mL ice-cold staining medium containing 1.5 μM propidium iodide. After 10min on ice, 40 μL of a stock solution of 50 mM PETG was added to stop β-Galactosidase activity. Cells were then analyzed using a Cytoflex LX (Beckman Coulter) flow cytometer and FlowJo v10.8.0 software (BD).

#### Multiplex assay for cytokine quantification

Multiplex assay was performed on ZART T cell supernatant using a 20-Plex ProcartaPlex Panel (Invitrogen) following manufacturer’s instructions. Alternatively, multiplex assay was performed on the supernatant of activated CD8^+^ T cells prepared by stimulating OT-I TCR T cells as described.^14^ Standards were reconstituted with Universal Assay Buffer diluted at 1:2 in assay diluent. For analyte capture, the plate was incubated overnight at 4°C under agitation on an orbital shaker. Analyses were performed using a Bio-Plex 200 system equipped with Bio-Plex Manager software (Bio-Rad).

### Quantification and statistical analysis

All statistical tests (one-way ANOVA) were performed using Prism v.9.2.0 (GraphPad). Data are expressed as mean ± SEM. ns, not significant; ∗∗p < 0.01; ∗∗∗p < 0.001.

## Data Availability

Original code and files for 3D printing have been deposited on GitHub (https://github.com/boussolab/TEMPO). Data reported in this paper will be shared by the lead contact upon request.
