# Supplementary material for: Integration of intermittent calcium signals in T cells revealed by temporally patterned optogenetics
Source: iScience. 2023 Jan 26;26(2):106068. doi: 10.1016/j.isci.2023.106068 (PMC9942117; doi:10.1016/j.isci.2023.106068)
Supplement: Document S1. Figures S1–S5 [file mmc1.pdf]

iScience, Volume *26*

Supplemental information

Integration of intermittent calcium signals in T cells revealed by temporally patterned optogenetics

Béatrice Corre, Yassine El Janati Elidrissi, Justine Duval, Mailys Quilhot, Gaëtan Lefebvre, Solène Ecomard, Fabrice Lemaître, Zacarias Garcia, Armelle Bohineust, Erica Russo, and Philippe Bousso

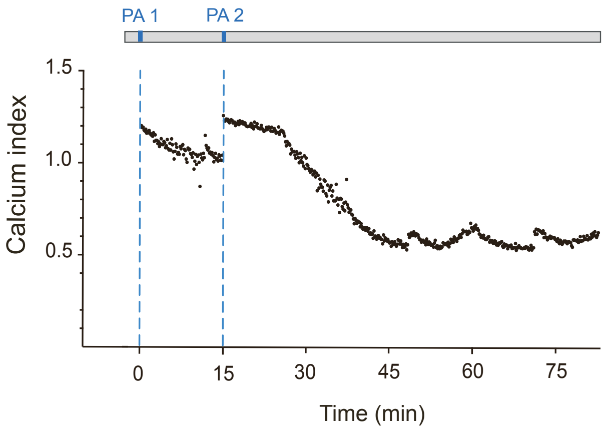

Figure S1. Monitoring the calcium response of ZART T cells during two photoactivations given 15 min apart (related to Figure 2). ZART T cells were subjected to two rounds of photoactivation (5s pulses) 15 min apart using TEMPO. Cell aliquots were collected at various time points after each photoactivation and analyzed by time-resolved flow cytometry to monitor calcium levels using the Twitch2B fluorescent reporter. Each cell aliquot was acquired for 15 min and immediately replaced by a new aliquot to generate an almost continuous signal curve by concatenation.

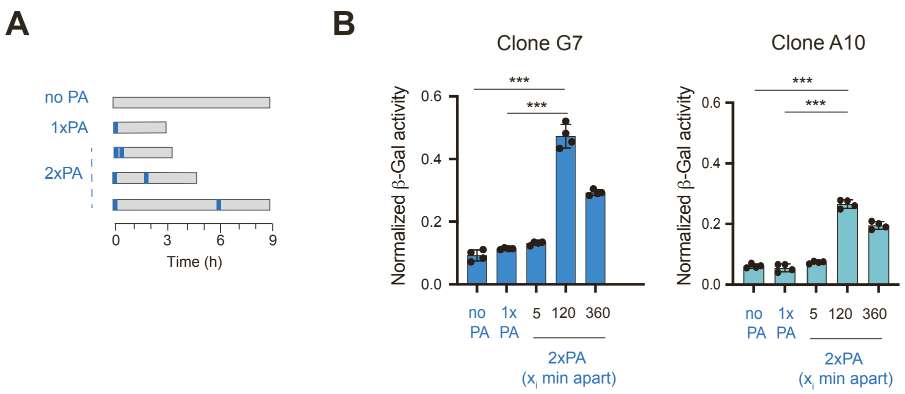

Figure S2. Integration of calcium signals in ZART T cells is also detected when analyzed at a fixed period (3h) after the last photoactivation (related to Figure 3). ZART T cells were subjected to TEMPO to evaluate their capacity to integrate intermittent calcium signals. Cells were exposed (or not) to one or two pulses (5s) of photoactivation delivered with the indicated time interval. For each condition, β–Galactosidase activity was quantified in the cell supernatant 3 hours after the last photoactivation. **A**) Experimental set-up illustrating the different sequences of photoactivation programmed in the TEMPO device. **B**) β–Galactosidase activity in cell lysates for the indicated photoactivation sequences. This assay was conducted on 2 independent ZART T cell clones (clone G7, clone A10).

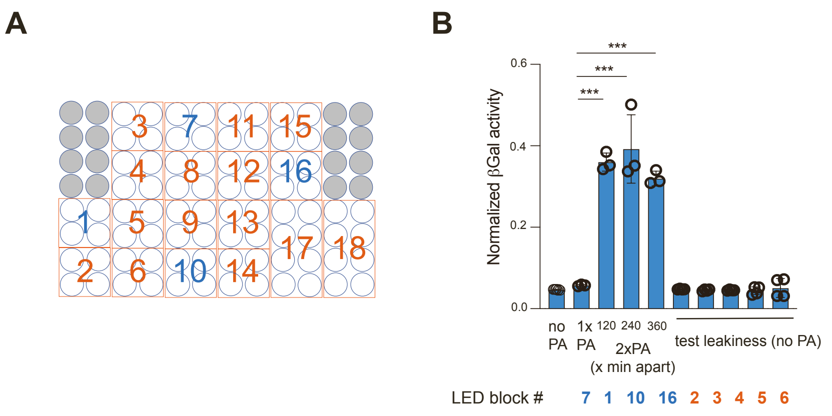

Figure S3. Lack of detectable optical signal leakage in the TEMPO device (related to Figure 3). To test the possibility that the LED of a given well may illuminate neighboring wells, we analyzed β-Gal activity in ZART T cells placed in non-illuminated wells situated near illuminated wells. **A**) Experimental set-up showing the illuminated wells (blocks #1, 7, 10, 16) and non-illuminated wells (blocks #2, 3, 4, 5, 6) **B**) Note that illuminated LED blocks #1, #10, #16 do not confer β-Gal activity in cells from neighboring wells (blocks #2-6).

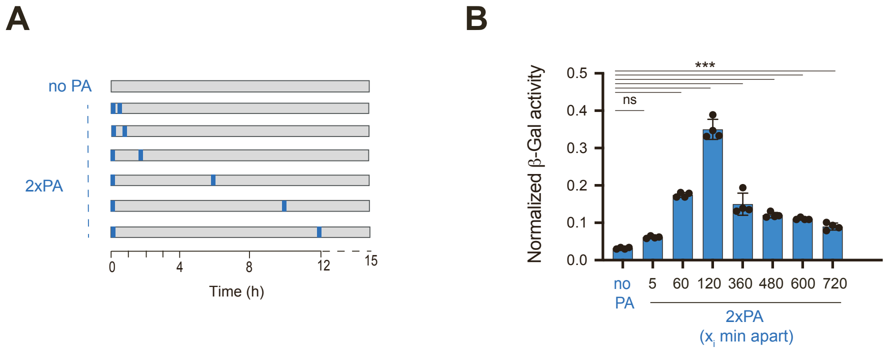

Figure S4. ZART T cells can integrate calcium signals separated by up to 12 hours (related to Figure 3).

ZART T cells were subjected to TEMPO to evaluate their capacity to integrate intermittent calcium signals. Cells were exposed (or not) to two pulses (5s) of photoactivation delivered with the indicated time interval (up to 12 hours apart). After 15h, β–Galactosidase activity was quantified in the cell supernatant. **A**) Experimental set-up illustrating the different sequences of photoactivation programmed in the TEMPO device. **B**) Bulk NFAT transcriptional responses were evaluated by monitoring β–Galactosidase activity in cell lysates for the indicated photoactivation sequences.

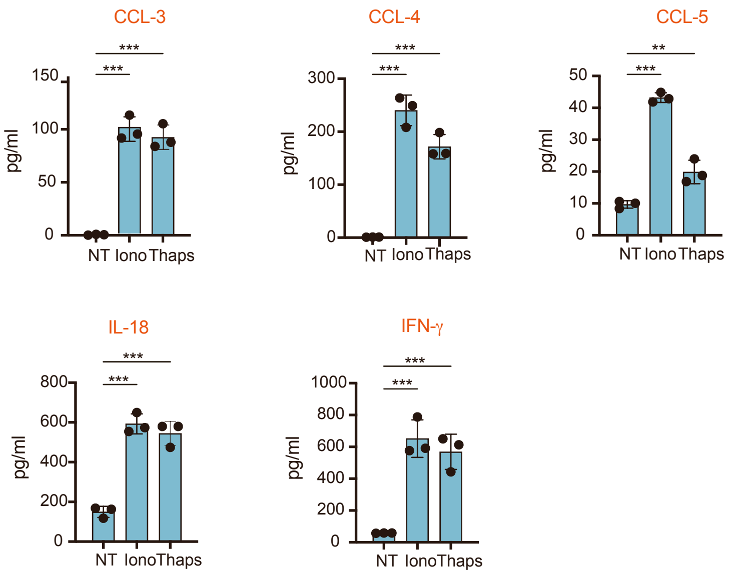

Figure S5. Primary effector CD8^{+} T cells can release cytokine/chemokine in response to calcium signals (related to Figure 4). CD8^{+} T cells were isolated from OT-I transgenic mice and stimulated with anti-CD3 and anti-CD28 mAb. Activated T cells were stimulated using the calcium ionophore ionomycin or using thapsigargin (an inhibitor of sarco endoplasmic reticulum Ca2 ATPase). After 6h, cell supernatants were collected and subjected to a multiplex cytokine assay. (NT, non treated; Iono, ionomycin; Thaps, thapsigargin).
